# Overexpression of a *Mycobacterium ulcerans* Ag85B-EsxH Fusion Protein in Recombinant BCG Improves Experimental Buruli Ulcer Vaccine Efficacy

**DOI:** 10.1371/journal.pntd.0005229

**Published:** 2016-12-12

**Authors:** Bryan E. Hart, Sunhee Lee

**Affiliations:** Human Vaccine Institute and Department of Medicine, Duke University Medical Center, Durham, North Carolina, United States of America; Swiss Tropical and Public Health Institute, SWITZERLAND

## Abstract

Buruli ulcer (BU) vaccine design faces similar challenges to those observed during development of prophylactic tuberculosis treatments. Multiple BU vaccine candidates, based upon *Mycobacterium bovis* BCG, altered *Mycobacterium ulcerans* (MU) cells, recombinant MU DNA, or MU protein prime-boosts, have shown promise by conferring transient protection to mice against the pathology of MU challenge. Recently, we have shown that a recombinant BCG vaccine expressing MU-Ag85A (BCG MU-Ag85A) displayed the highest level of protection to date, by significantly extending the survival time of MU challenged mice compared to BCG vaccination alone. Here we describe the generation, immunogenicity testing, and evaluation of protection conferred by a recombinant BCG strain which overexpresses a fusion of two alternative MU antigens, Ag85B and the MU ortholog of tuberculosis TB10.4, EsxH. Vaccination with BCG MU-Ag85B-EsxH induces proliferation of Ag85 specific CD4^+^ T cells in greater numbers than BCG or BCG MU-Ag85A and produces IFNγ^+^ splenocytes responsive to whole MU and recombinant antigens. In addition, anti-Ag85A and Ag85B IgG humoral responses are significantly enhanced after administration of the fusion vaccine compared to BCG or BCG MU-Ag85A. Finally, mice challenged with MU following a single subcutaneous vaccination with BCG MU-Ag85B-EsxH display significantly less bacterial burden at 6 and 12 weeks post-infection, reduced histopathological tissue damage, and significantly longer survival times compared to vaccination with either BCG or BCG MU-Ag85A. These results further support the potential of BCG as a foundation for BU vaccine design, whereby discovery and recombinant expression of novel immunogenic antigens could lead to greater anti-MU efficacy using this highly safe and ubiquitous vaccine.

## Author Summary

*Mycobacterium ulcerans* (MU) infection causes a highly disfiguring, necrotic skin disease known as Buruli ulcer (BU). Antibiotic treatments have low efficacy if the infection is diagnosed after ulceration begins, leading to frequent dependence on surgical removal of infected tissues. A prophylactic vaccine for BU does not exist and several attempts to create an effective vaccine have shown limited success. We recently demonstrated that a recombinant strain of *M*. *bovis* BCG expressing the immunodominant MU-Ag85A conferred significantly enhanced protection against experimental BU compared to the standard BCG vaccine. Here we show that BCG expression of a fusion between two alternative MU antigens, Ag85B and EsxH, can promote antigen-specific T cell and humoral immune response capable of significantly improving survival and protection against BU pathology, compared to BCG MU-Ag85A alone. These results support the potential for using the highly safe and ubiquitous BCG vaccine as a platform for further BU vaccine development.

## Introduction

Subcutaneous skin infection by *Mycobacterium ulcerans* (MU) leads to a potentially disfiguring, necrotic condition known as Buruli ulcer (BU) [1]. What often begins as an indolent skin nodule or small lesion can ultimately progress to expanding ulcerations, body-wide scarring, loss of limbs or eyes, and osteomyelitis [2]. These infections disproportionately affect children and are largely endemic to Sub-Saharan Africa, Australia, and Japan, where the unconfirmed mode of transmission is thought to be dependent on exposure to contaminated wetland areas and insect vectors [3, 4]. Treatment regimens include lengthy combination anti-mycobacterial therapies, however, lack of medical access, absence of rapid and accurate diagnostics, and the often misleading symptoms of BU frequently lead to significant delays in therapeutic action [5, 6]. At the point of extensive tissue damage, surgical debridement and skin grafting is required, resulting in significant morbidity and social stigmatization [7, 8]. Antibiotics can be effective against MU if administered at an early time point prior to ulceration, and side effects of treatment can include nephrotoxicity and hearing loss [9]. While there is increasing promise for less toxic antibiotic therapies, currently no prophylactic vaccine is available to prevent BU in the areas with greatest prevalence [10].

BU vaccine research strategies have largely focused on prime-boost regimens using recombinant DNA and MU proteins, however, the efficacy of these approaches has not surpassed the transient, cross-reactive protection observed during experimental vaccination with tuberculosis vaccine strain, *Mycobacterium bovis* bacillus Calmette- Guérin (BCG) [11–18]. BCG, the most ubiquitous World Health Organization-approved vaccine administered across the world, possesses a promising safety profile but low efficacy against pulmonary tuberculosis afflicting millions of people [19, 20]. Experimental BCG vaccination has been studied using BU animal models and has been shown to confer protection by delaying ulceration after murine footpad challenge with MU [11]. While BCG vaccination extends the time to appreciable footpad swelling, protection ultimately wanes and animal euthanasia is required. Retrospective studies in humans also provide support for the potential use of BCG as a foundation for an effective BU vaccine. Patients previously vaccinated with BCG were shown to have delayed onset to ulceration after infection with MU, as well as significant protection against developing complications of MU infection, such as osteomyelitis [21–23]. These lines of evidence further support the potential of BCG as a foundation for BU vaccine design, whereby improvement of BCG immunogenicity could lead to greater efficacy using this highly safe and ubiquitous vaccine.

BCG has previously been engineered to express various recombinant immunogenic antigens and protein fusions for use in TB vaccine development, with numerous observed *in vivo* effects [24–26]. Recombinant BCG vaccine strains which have been engineered to overexpress major antigenic secretory proteins, such as ESAT-6, TB10.4, CFP10, heat shock proteins, and members of the mycolyl transferase complex Ag85A, Ag85B, and Ag85C, have displayed the greatest promise by increasing both humoral IgG antibody production and CD4 mediated T_h_1 responses against *M*. *tuberculosis* challenge [27–33]. Similar strategies have been investigated in application to BU vaccine design as well, with varying degrees of success. Priming with a DNA-based vaccine encoding multiple MU polyketide synthase modules and boosting with recombinant protein by Roupie *et al*. yielded differential levels of antigen-specific IgG responses, as well as IFNγ and IL-2 secretion upon recombinant MU antigen stimulation [12]. However, no improvement in protection over the level conferred by BCG vaccination was observed. Alternatively, an investigation by Tanghe *et al*. used a similar strategy that employed the plasmid-based expression of MU Ag85A as a prime followed by a recombinant protein boost [13, 14]. This vaccination regimen produced appreciable antigen-specific immunogenicity which correlated with a level of protection against MU challenge that was similar to that achieved by BCG vaccination alone.

We recently utilized a combination of strategies from the TB vaccine field, as well as those used by previous attempts to design anti-MU vaccines by engineering a quality controlled recombinant strain of BCG that overexpressed MU-Ag85A [15]. In this study, we showed that not only could this vaccine strain significantly induce proliferation of antigen-specific CD4^+^ T cells and increase IFNγ^+^ Th_1_ splenocytes responsive to whole MU and subcellular fractions, but subcutaneous priming also decreased MU burden, protected against mycolactone-induced pathology, and extended the lifespan of MU-challenged mice to significantly greater levels compared with BCG vaccination alone. Knowing that overexpression of one MU antigen by BCG could have these effects, we were subsequently interested in determining if alternative antigens or combinations of antigens could yield improvements on vaccine immunogenicity and efficacy.

The immunodominant antigens, Ag85B and TB10.4, are two such antigens that have been successfully used to augment the protective qualities of BCG against experimental tuberculosis in animal models [29, 31, 32, 34–37]. These individual antigens, as well as fusion proteins combining various small antigens or important T cell epitopes from multiple antigens, have also been successfully expressed heterologously in BCG. Importantly, these constructs have been shown to initiate production of antigen-specific CD4^+^ T cell populations known to be vital in generating the same anti-mycobacterial Th_1_ responses hypothesized to play a role in containment of MU in humans. Due to the encouraging results from our previous study involving BCG expression of Ag85A and the large body of evidence supporting the usefulness of Ag85B and TB10.4 antigens against other mycobacterial diseases, we generated a vaccine strain of BCG which expressed a fusion protein combining MU-Ag85B and the TB10.4 homolog from *M*. *ulcerans*, MU-EsxH (BCG MU-Ag85B-EsxH).

Here we will show that, compared to BCG vaccination, a single subcutaneous dose of BCG MU-Ag85B-EsxH induced significantly enhanced antigen-specific humoral responses, CD4^+^ T cell proliferation, and T_h_1 splenocyte responses in mice. In addition, a single, un-boosted, subcutaneous dose of BCG MU-Ag85B-EsxH conferred significantly greater protection compared to BCG by reducing bacterial burden in MU challenged footpads, resisting pathologies associated with MU infection, and significantly lengthened the lifespan of MU-challenged mice. Importantly, these effects were statistically improved over those conferred by BCG MU-Ag85A, which was the first and only vaccine strain superior to BCG vaccination against BU in mice.

## Methods

### Mice and ethics statement

Female, 6–8 week old, C57BL/6 mice were obtained from Jackson Laboratories. These mice were 14–16 weeks old by time of footpad challenge with MU. Animal work was approved by the Duke University Institutional Animal Care and Use Committee (IACUCU protocol A065-13-03). IACUC protocols performed at Duke University adhered to the AAALAC, USDA, Guide for Care and Use of Laboratory Animals and Public Health Service Policy on Humane Care and Use of Laboratory Animals and Animal Welfare Act.

### Bacterial strains and culture

All strains of *Mycobacterium bovis* BCG-Danish (BCGD) were cultured at 37°C on solid Difco Middlebrook 7H10 agar or in liquid Difco Middlebrook 7H9 media supplemented with 0.5% glycerol, oleic-albumin-dextrose-catalase (OADC), and 0.05% tyloxapol. Selection of BCG transformants expressing MU Ag85B-EsxH was accomplished by adding 25 or 50 μg/ml hygromycin to liquid or solid media, respectively. Liquid cultures consisting of volumes less than 50 ml were shaken at 120 rpm and larger volumes were expanded to 250 ml or less in one liter bottles rotated at 6 rpm. High-volume vaccine accession lots were aliquoted into 1 ml cryovials and frozen at a concentration of OD_600_ 1 (~10^8^ CFU/ml). Virulent *M*. *ulcerans* 1615 was kindly provided by Dr. Pamela Small (University of Tennessee) and was cultured at 32°C in Middlebrook media as prepared for BCG. For purification of plasmid DNA and sequencing, DH5α *Escherichia coli* (*E*. *coli*) was grown on lysogeny broth (LB) agar plates or in LB supplemented with 50 μg/ml hygromycin.

### Plasmid constructions and transformation

The *M*. *ulcerans* Ag85B open reading frame and endogenous secretion signal were amplified from MU1615 genomic DNA and cloned into the mycobacterial vector pMV261 [38], where antigen expression was mediated by the constitutive mycobacterial *hsp60* promoter (henceforth, pSL402). Selection of plasmids was controlled through hygromycin resistance. The influenza hemagglutinin (HA) epitope was added to the C-terminus of the EsxH fusion. Electrocompetent BCG cells were prepared by centrifuging log phase culture (OD_600_ 0.6–0.8) at 3000 rpm for 10 minutes, followed by washing in a buffer of 10% glycerol and 0.05% tyloxapol. Mycobacteria electroporated with 0.5 μg plasmid DNA were recovered by shaking at 37°C in 1 ml Middlebrook 7H9 media overnight.

### Quality control of vaccine lots

A series of quality control characterizations was performed on vaccine accession lots by assessing expression of recombinant antigen, contamination, and plasmid DNA retention as previously described [39]. Bacterial lysates for immunoblot were prepared by centrifuging 10 ml of log-phase liquid culture at 3000 rpm for five minutes. After washing in phosphate buffer saline + 0.05% tyloxapol (PBST), the final cell pellet was resuspended in 200 μl lysis buffer with glass beads and vortexed for three minutes. Lysates were clarified by collecting the supernatant after centrifugation for five minutes at 3000 rpm. Pre-cast SDS PAGE gels were loaded with 15 μl clarified lysate boiled in Laemmli buffer and run for one hour at 130V. Protein was then transferred to PVDF membranes for one hour at 30V. Blocking of membranes was performed by shaking in 5% fat free milk in TBS with 0.1% tween (TBST) for one hour at room temperature. For detection of the HA epitope, mouse anti-HA-HRP (clone 3F10, Roche) was diluted in 5% milk-TBST (1:1000) and incubated room temperature for one hour. After washing in TBST, detection of proteins was performed using chemiluminescence (Lumi-light, Roche). Plasmid DNA was purified using a modified Qiagen Miniprep protocol [39]. Isolated plasmids were heat-shocked into *E*. *coli* DH5α and plasmid DNA was re-purified from the resulting transformants. Correctly sized plasmid inserts were assessed by reaction with NdeI/EcoRV and analysis using gel electrophoresis. Plasmid inserts from 10 *E*. *coli* clones were sequenced and analyzed using Clone Manager software (Sci-Ed). Vaccine contamination was assessed by spread plating 100 μl of thawed accession lot material on chocolate agar and examining for growth following a two week incubation at 37°C.

### Mouse vaccination and infection

For protection studies, C57BL/6 mice were subcutaneously vaccinated by injection of 100 μl (~10^7^ cells) from an accession lot vial of empty-vector BCG (pHA), recombinant BCG MU-Ag85A, or BCG MU-Ag85B-EsxH into the scruff of the neck. Eight weeks post-vaccination, the mice were challenged intradermally with 10^5^ *M*. *ulcerans* 1615 (MU1615) via the footpad. MU1615 challenge inocula were consistently accessed from the same accession lot frozen at -80°C and were tested for virulence by pathology in mouse footpad models. The width and height of footpad swelling from infected mice were measured at two to three week intervals using digital calipers. To reduce animal suffering and in compliance with IACUC protocol, infected mice were euthanized once footpad swelling height exceeded 4.5 mm, prior to any visible ulceration.

### T cell flow cytometry

Quantitation of Ag85-specific CD4^+^ T cell levels was performed by flow cytometric analysis of MHCII tetramer staining. Mice were inoculated subcutaneously or intravenously (via the retro-orbital route) with 100 μl (~10^7^ cells) of a freshly thawed vaccine accession lot vial. Weekly blood samples were collected retro-orbitally followed by isolation of peripheral blood mononuclear cells (PBMCs) by gradient centrifugation using Lympholyte M (Cedarlane). PBMC buffy coats were collected, washed in 10 ml phosphate buffered saline (PBS), and were resuspended in 2 ml of ACK lysis buffer to remove erythrocytes. After centrifugation at 2000 rpm, PBMC pellets were washed with PBS and stained with APC-conjugated *M*. *tuberculosis* Ag85B-MHCII tetramer (1:500, NIH Tetramer Core Facility) in flow buffer (2% fetal bovine serum in PBS). The 15 amino acid epitope, FQDAYNAAGGHNAVF, was recognized by this tetramer and shares high sequence homology with MU Ag85. PBMCs were stained with tetramer for 30 minutes at 37°C and then stained with FITC-conjugated anti-mouse CD4 (1:500, clone GK-1.5, Biolegend) and PE-Cy5 anti-mouse CD8 (1:200, clone 53–6.7, Biolegend) for 30 minutes on ice. The PBMCs were then washed in with flow buffer, centrifuged for 2000 rpm for five minutes, and were then resuspended in 4% paraformaldehyde. Following fixation cells underwent flow cytometric analysis using a Becton Dickinson (BD) LSRII and FlowJo software (Tree Star Inc.). To stain effector and central memory cells, the tetramer protocol was followed by additional antibody incubation steps with APC-Cy7 anti-mouse CD4 (clone GK-1.5), FITC anti-mouse CD62L (clone MEL-14, BD Pharmingen), and PE-Cy7 anti-mouse CD44 (clone IM7, BD Pharmingen).

### Acid-fast microscopy

MU bacilli present in infected footpads were quantified using a method previously described [15]. At 6 and 12 weeks post-challenge, footpads infected with MU1615 were removed from euthanized mice and for disinfection and dissection. The footpads were disinfected by five-minute contact with 70% ethanol, followed by three washes in PBST. The challenged footpads were then minced and crushed by mortar and pestle. Resulting homogenates were subjected to N-acetyl-L-cysteine (NALC)/NaOH treatment by adding a mixture (50:50) of 4% NaOH and 2.9% sodium citrate + 1% NALC for 20 minutes. Homogenates were pelleted at 3000 rpm for five minutes, washed with PBST, and larger particulates were removed by passage through a 40 μm mesh. 5 μl of filtrate was then evenly distributed within a 0.8 cm^2^ circle upon glass microscope slides. Heat-fixed smears were stained with auramine-rhodamine (BD Biosciences), destained with acid alcohol, and counterstained with potassium permanganate. Stained slides were viewed under 100x oil immersion using a Nikon X microscope. Acid-fast bacilli (AFB) were counted in four random fields of view (FOV) per animal (16 images total per vaccinated group). Total AFB calculations were performed by multiplying cell counts by the number of 0.038 mm^2^ FOVs in each marked smear area per microliter of applied filtrate.

### IFNγ ELISPOT

ELISPOT plates (PVDF, 96 well) were equilibrated with 70% ethanol, washed with PBS, and were coated overnight with anti-mouse IFNγ antibody (1 μg/ml, clone AN18, Mabtech). Necropsies were performed on mice eight weeks following intravenous vaccination to harvest splenocytes for growth in RPMI complete media (RPMI with L-glutamine and 10% fetal bovine serum). 96 well plates were blocked using RPMI media and 6 x 10^5^ splenocytes were combined in each well with varied agonists: MU-Ag85A peptide (100 μg/ml, FQAAYNAAGGHNAVWNFDDN), MU-Ag85B peptide (100 μg/ml, FQDAYNAAGGHNAVFNFNDN), heat killed MU (HKMU, 1 mg/ml), or whole cell lysate prepared from log phase MU liquid culture. Splenocytes were stimulated for 16 hours at 37°C. The plates were then washed with PBS + 0.05% tween 20 followed by a two hour incubation with secondary anti-mouse IFNγ antibody (1:1000, clone R46A2, Mabtech) at 37°C. After a wash and three hour room temperature incubation with VectaStain avidin peroxidase complex (Vector Labs), plates were incubated with 3-amino-9-ethylcarbazole substrate for five minutes. The reaction was ceased by submersion of plates in deionized water. Spots were visualized and quantified using a CTL Immunospot plate reader.

### IgG ELISA

High-binding, 384-well plates (Corning) were coated overnight at 4°C with 100 ng/ml recombinant *M*. *tuberculosis* Ag85A, Ag85B, or purified Ag85 complex (BEI resources, NIAID, NIH) diluted in 0.1 M sodium bicarbonate. Wells were then washed once, blocked with 40 μl blocking buffer (4% whey protein, 15% goat serum, 0.5% tween 20, and 0.05% sodium azide in PBS) for one hour at room temperature, and then washed again. After blocking, of a 1:100 dilution of intravenously vaccinated mouse serum in blocking buffer was added for 2 hours at room temperature. Plates were washed four more times and 15 μl of a 1:1000 dilution of goat anti-mouse IgG (Southern Biotech 1030–05) was added for one hour at room temperature. After four further washes, 20 μl of tetramethylbenzidine substrate was added per well for up to 15 minutes. The reaction was stopped by addition of 20 μl 0.33 N HCL solution and absorbance was read at 450 nm.

## Results

### Expression of MU-Ag85B-EsxH fusion in recombinant BCG

**Fig 1:**
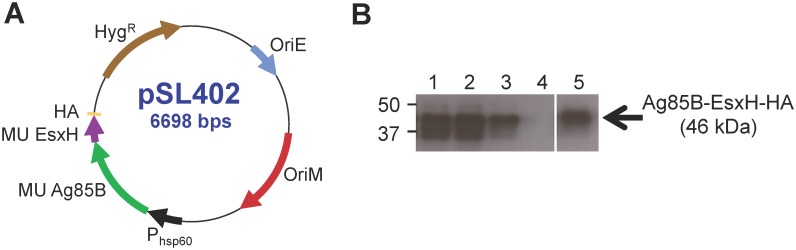
Heterologous expression of the MU-Ag85B-EsxH fusion protein by *M*. *bovis* BCG. **A**. The 6698 base pair (bp) plasmid map for pSL402 is shown. Constitutive expression of MU-Ag85B-EsxH (green, purple) C-terminally fused to an HA epitope tag (yellow) is driven by the promoter region from *hsp60* (black). Plasmid replication is regulated in in mycobacteria using *oriM* (red) and in *E*. *coli* shuttle vectors using *oriE* (blue). Plasmid retention is selected by a hygromycin resistance cassette (brown). **B**. Three vaccine accession lot vials of BCG transformed with pSL402 were chosen at random for Western analysis of MU-Ag85B-EsxH expression using anti-HA antibody (lanes 1–3). The expected band size was 46 kDa. The negative control was a lysate from BCG transformed with an empty expression vector (lane 4) and the positive control was a lysate from *M*. *smegmatis* transformed with pSL402 (lane 5).

We have previously described the generation of quality controlled accession lots of recombinant BCG expressing antigens of interest, particularly MU-Ag85A [39]. In order to generate a vaccine strain of BCG which expressed an in-frame fusion between MU-Ag85B and the *M*. *tuberculosis* TB10.4 homolog, MU-EsxH, electrocompetent BCG was transformed with pSL402 (Fig 1A). The pSL402 replicating plasmid controls transcription of MU-Ag85B-EsxH containing a C-terminal fusion to the influenza hemagglutinin epitope using the constitutive mycobacterial *hsp60* promoter. Plasmid replication was regulated by the mycobacterial *oriM* and by *oriE* in *E*. *coli* shuttle strains. Selection of bacterial transformants utilized plasmid-encoded resistance to hygromycin. Fig 1B displays expression patterns of the MU-Ag85B-EsxH fusion protein in whole cell lysates from 3 randomly picked BCG transformants. A single transformant which strongly expressed the fusion antigen was selected to produce a large-volume vaccine accession lot upon which a quality control panel was employed to further characterize expression of recombinant antigen, purity of the vaccine lot, and integrity of the recombinant plasmid sequence [39].

### BCG MU-Ag85B-EsxH vaccination induces proliferation of antigen-specific CD4^+^ T cells

Anti-mycobacterial immunity is largely governed by responses from CD4^+^ T helper cells [40, 41]. In order to determine if antigen-specific adaptive immune responses could be generated by vaccination with BCG-MU-Ag85B-EsxH, C57BL/6 mice were either subcutaneously or intravenously or primed with 10^7^ bacilli from thawed quality controlled vaccine lots prepared as previously described [39]. At weekly intervals, retro-orbital blood samples were collected to isolate peripheral blood mononuclear cells. Flow cytometric analysis of staining by MHCII tetramer was subsequently used to quantify the percentage of CD4^+^ T cells which recognized the Ag85 epitope, FQDAYNAAGGHNAVF.

**Fig 2:**
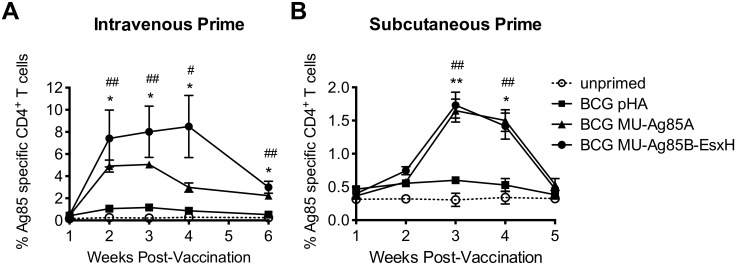
BCG MU-Ag85B-EsxH vaccination induces proliferation of antigen-specific CD4^+^ T cells. C57BL/6 mice remained unprimed (dotted black, open circle) or were intravenously primed (**A**) or subcutaneously primed (**B**) with 10^7^ BCG transformed with empty-vector (pHA, square), the MU-Ag85A pSL401 expression vector (triangle), or with the MU-Ag85B-EsxH pSL402 vector (circle). At weekly time points, retro-orbital blood was collected for T lymphocyte isolation. Flow cytometric analysis was performed to quantify levels of CD4^+^ T cells bound to MHCII-Ag85 tetramer. Asterisks and number signs indicate statistical analysis by the student’s t-test (n = 5 for each group) comparing the empty-vector BCG group to BCG MU-Ag85B-EsxH and BCG MU-Ag85A groups, respectively. Error bars represent standard deviation. *p<0.05, **p<0.002, #p<0.05, ##p<0.001.

Fig 2A and 2B show the levels of antigen-specific T helper cells induced by intravenous or subcutaneous vaccination, respectively. Responses from BCG MU-Ag85B-EsxH were compared to those from mice vaccinated with BCG containing an empty expression vector, BCG MU-Ag85A, and unprimed mice. While low levels of Ag85-specific T cells were induced in response to endogenously expressed antigen in BCG, a significantly greater number of T cells was produced upon vaccination with BCG overexpressing Ag85A or Ag85B-EsxH. The vaccination route did differentially affect the speed and amplitude with which peak responses were reached. Intravenous inoculation induced quicker, larger, and more prolonged T cell responses compared to the subcutaneous route; an 8% Ag85-specific T helper cell population was reached at 3 weeks after the intravenous injection with BCG MU-Ag85B-EsxH compared to a 1.75% peak response post-subcutaneous vaccination. Greater statistical significance was also achieved between BCG Ag85B-EsxH versus empty-vector BCG compared to the enhancement of BCG MU-Ag85A responses during the intravenous injection at weeks 2 and 3 post-vaccination (p<0.05, and p<0.001, respectively). Interestingly, the peak response to BCG MU-Ag85A reached a maximum of 5% compared to the 8% peak following vaccination with BCG MU-Ag85B-EsxH. However, subcutaneous vaccination resulted in highly similar T cell proliferative responses between BCG MU-Ag85A and BCG MU-Ag85B-EsxH, both of which were significantly higher than those induced by empty-vector BCG (p<0.01, p<0.002).

Additionally, we attempted to use MHCII tetramer staining to detect T cell populations capable of recognizing the *M*. *tuberculosis* TB10.4 epitope, SSTHEANTMAMMARDT (data not shown). However, no tetramer positive populations were detected, which may be due to the presence of two amino acid substitutions at this tetramer peptide position within the MU-EsxH sequence. Together, these data suggest that like BCG MU-Ag85A, BCG MU-Ag85B-EsxH is capable of generating helper T cell populations responsive to Ag85, an immunodominant antigen previously demonstrated to play a role in vaccine-mediated protection against MU.

### BCG MU-Ag85B-EsxH induces replication of antigen-specific CD4^+^ central and effector memory T cells

**Fig 3:**
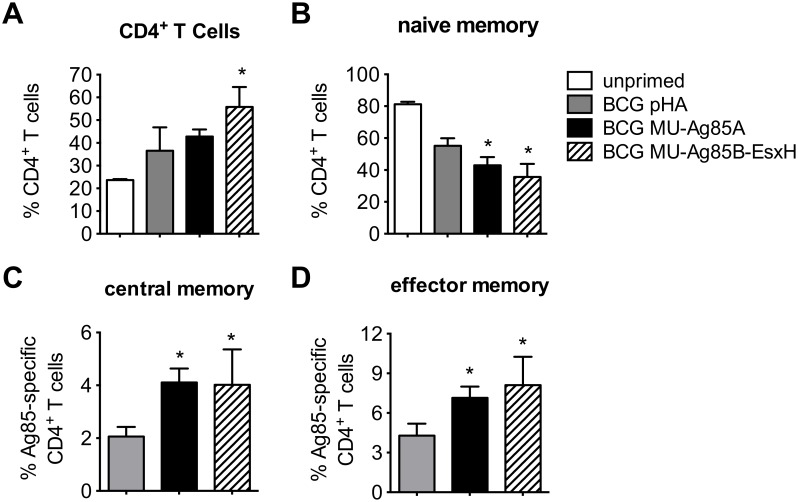
BCG MU-Ag85B-EsxH induces replication of antigen-specific CD4^+^ effector and central memory T cells. At 4 weeks post-prime with empty-vector BCG (gray), BCG MU-Ag85A (black), or BCG MU-Ag85B-EsxH (striped), mice were bled retro-orbitally and peripheral leukocytes were stained CD4 and the memory markers CD62L and CD44. Percentages of total CD4^+^ T cells (A) and CD62L^lo^CD44^lo^ naïve CD4^+^ T cells (B) were quantified, as were antigen-specific effector memory CD4^+^ T cells (CD62L^lo^CD44^hi^) and central memory CD4^+^ T cells (CD62L^hi^CD44^hi^) which specifically bound Ag85-MHCII-tetramer (C, D). Asterisks indicate statistical analysis by the student’s t-test (n = 4 for each group). Error bars represent standard deviation. *p<0.05.

Evidence from previous studies has highlighted the importance of CD4^+^ memory T cell populations in establishing greater efficacy for mycobacterial vaccines [42]. To quantify the levels of memory T cells produced by vaccination with the recombinant BCG strains, C57BL/6 mice were intravenously primed with 10^7^ bacilli and, four weeks later, peripheral lymphocyte staining was performed for CD4 and the CD62L and CD44 memory T cell markers. While the absolute numbers of CD4^+^ T cells rose in all vaccinated groups regardless of BCG strain, vaccination with BCG MU-Ag85B-EsxH induced significantly higher levels compared to BCG (Fig 3A, p<0.05). Of those populations, naïve T cells were significantly reduced upon vaccination with either BCG MU-Ag85A or BCG MU-Ag85B-EsxH (Fig 3B, p<0.05). Interestingly, both Ag85-specific CD4^+^ effector memory and central memory T cell populations were significantly higher upon vaccination with either BCG MU-Ag85A or BCG MU-Ag85B-EsxH compared to BCG alone (Fig 3C and 3D, p<0.05). These data suggest that BCG MU-Ag85B-EsxH could also be useful in establishing T cell memory reservoirs capable of recognizing an MU antigen known to be immunoprotective and possibly representing sources of anti-mycobacterial IFNγ and IL-2 [43].

### Expression of MU-Ag85B-EsxH enhances Th_1_ responsiveness to *M*. *ulcerans* antigens compared to BCG

**Fig 4:**
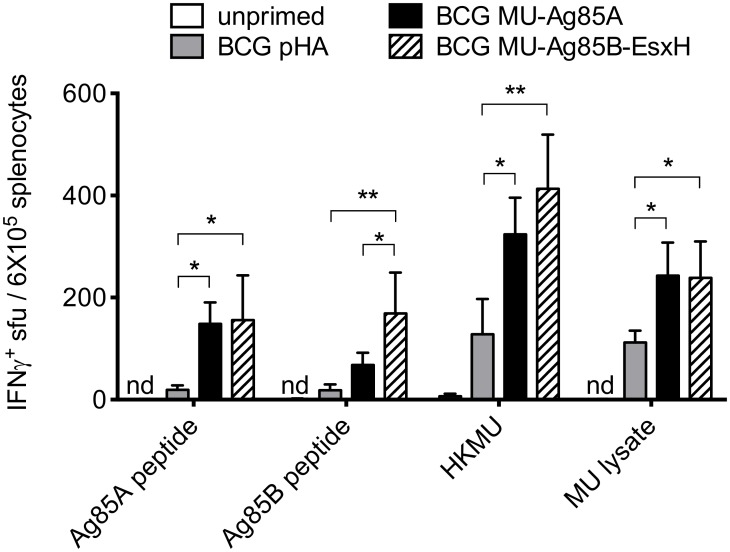
Expression of MU antigens increases responsiveness of T_h_1 cells to *M*. *ulcerans* and purified components. C57BL/6 mice remained unprimed (white) or were intravenously primed with 10^7^ BCG transformed with empty-vector (gray), the MU-Ag85A pSL401 expression vector (black), or with the MU-Ag85B-EsxH pSL402 vector (striped). At 8 weeks post-prime, mice were euthanized and splenocytes were isolated for stimulation with MU-Ag85A peptide, MU-Ag85B peptide, whole heat killed MU1615 (HKMU), or MU whole cell lysate. ELISPOT spot forming units (SFU) were used to quantify the number of IFNγ-producing cells after 24 hours of stimulation. Asterisks indicate statistical analysis by the student’s t-test (n = 5 for each group). Error bars represent standard deviation. *p<0.05, **p<0.01.

The requirement of IFNγ-yielding Th_1_ responses for generating efficacious anti-mycobacterial immunity has been characterized by several studies [44]. To determine if production of antigen-specific T cells following vaccination with recombinant BCG strains could generate such responses, C57BL/6 mice were primed with empty-vector BCG, BCG MU-Ag85A, or BCG MU-Ag85B-EsxH. Eight weeks post-vaccination, mice were euthanized and harvested splenocytes *were* stimulated with various MU antigens: MU-Ag85A peptide, MU-Ag85B peptide, heat-killed MU1615 (HKMU), or MU whole cell lysate. Quantification of IFNγ-producing splenocytes was performed by enzyme-linked immunospot (ELISPOT) and the numbers of IFNγ^+^ spot-forming units (SFU) detected after 24 hours of agonist stimulation were calculated (Fig 4).

Vaccination with both BCG MU-Ag85A and BCG MU-Ag85B-EsxH yielded significantly increased IFNγ^+^ splenocytes compared to BCG alone when stimulated with all MU antigens (p<0.05). The greatest responses were detected upon stimulation with whole heat-killed MU, whereby priming with BCG MU-Ag85A and BCG MU-Ag85B-EsxH increased SFU over BCG pHA by 2.8-fold and 3.3-fold, respectively. Notably, cytokine-secreting splenocyte numbers trended higher in the BCG MU-Ag85B-EsxH-vaccinated mice compared to BCG MU-Ag85A following stimulation with MU-Ag85B peptide (>2-fold increase) and whole heat killed MU (1.2-fold increase). Four separate *M*. *tuberculosis* TB10.4 peptides were also used to stimulate splenocytes, but no appreciable IFNγ^+^ populations were detected. Interestingly, the response of BCG MU-Ag85B-EsxH vaccinated mice to HKMU was over double that of the SFU generated by stimulation with MU-Ag85B peptide alone. These data suggest that the BCG MU-Ag85-EsxH vaccine may increase responsiveness of functional Th_1_ cells to additional Ag85B peptides or to other antigens expressed by *M*. *ulcerans* cells.

### Ag85-specific humoral responses are induced by BCG MU-Ag85B-EsxH

**Fig 5:**
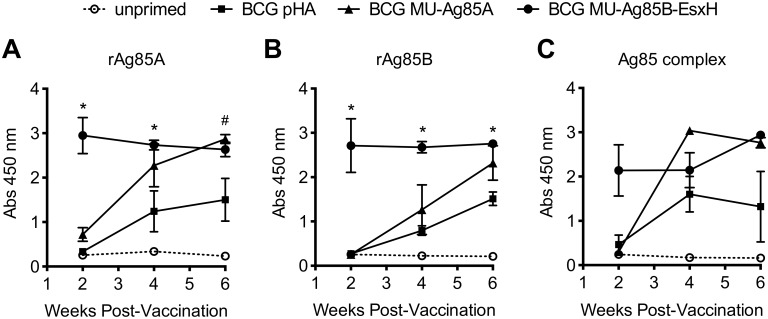
Ag85-specific humoral responses are induced by BCG MU-Ag85B-EsxH. C57BL/6 mice remained unprimed (dotted, open circle) or were intravenously primed with 10^7^ BCG transformed with empty-vector (square), the MU-Ag85A pSL401 expression vector (triangle), or with the MU-Ag85B-EsxH pSL402 vector (circle). At 2, 4, and 6 weeks post-vaccination peripheral submandibular blood was collected for serum isolation and IgG ELISA against recombinant M. tuberculosis Ag85A (rAg85A, **A**), recombinant M. tuberculosis Ag85B (rAg85B, **B**), or purified M. tuberculosis Ag85 complex proteins (**C**). Asterisks and number signs indicate statistical analysis by the student’s t-test (n = 5 for each group) comparing the empty-vector BCG group to BCG MU-Ag85B-EsxH or BCG MU-Ag85A groups, respectively. Error bars represent standard deviation. *p<0.05, #p<0.05.

Humoral responses to mycobacterial infection are becoming increasingly recognized in adaptive defense and as potential therapeutics [45–47]. To determine if use of recombinant BCG could induce antigen specific antibody responses *in vivo*, C57Bl/6 mice were vaccinated with empty-vector BCG, BCG MU-Ag85A, or BCG MU-Ag85B-EsxH and peripheral blood was collected biweekly for 6 weeks. Sera were subsequently isolated and tested for antibodies specific to immunogenic Ag85 proteins by IgG ELISA. Fig 5A, 5B and 5C display time course antibody responses to recombinant *M*. *tuberculosis* Ag85A, recombinant Ag85B, and purified Ag85 complex, respectively. At 2 weeks post-vaccination, all IgG responses were low in mice vaccinated with BCG or BCG MU-Ag85A; however, BCG MU-Ag85B-EsxH induced high levels of anti-Ag85A, Ag85B, and Ag85 complex IgG. Over the course of 6 weeks, antibody kinetics varied depending on antigen and vaccine group. Antibodies produced by BCG alone were consistently lower than those elicited by recombinant strains but did increase over time. Ag85A IgG responses from BCG MU-Ag85A-vaccinated mice continued to rise over time compared to the BCG MU-Ag85B-EsxH response which began high and slightly decreased over 6 weeks. A similar trend was observed for BCG MU-Ag85A induced anti-Ag85B responses, however, BCG MU-Ag85B-EsxH vaccination yielded a very high response which did not decline over this time course. Finally, IgG antibody induction against purified Ag85 complex peaked for BCG MU-Ag85A-vaccinated mice at 4 weeks but began to decline by 6 weeks. Conversely, anti-Ag85 complex responses induced by BCG MU-Ag85B-EsxH immediately began high and continued to climb by week 6. Compared to empty-vector BCG, BCG MU-Ag85A vaccination did not generate statistically significantly higher IgG responses for any antigens except to rAg85A at week 6. However, BCG MU-Ag85B antibody induction was statistically significantly higher (p<0.05) over empty-vector BCG for both rAg85A and rAg85B for all time points except anti-rAg85A at week 6. Additionally, recombinant *M*. *tuberculosis* TB10.4 was plated in the same ELISA format, however no IgG could be detected by ELISA. Together these data highlight the ability of BCG MU-Ag85B-EsxH to induce high levels of IgG reactive to multiple immunogenic Ag85 antigens, with a more rapid initial response compared to BCG or BCG MU-Ag85A.

### BCG MU-Ag85B-EsxH vaccination is significantly more protective than BCG or BCG MU-Ag85A against MU challenge

**Fig 6:**
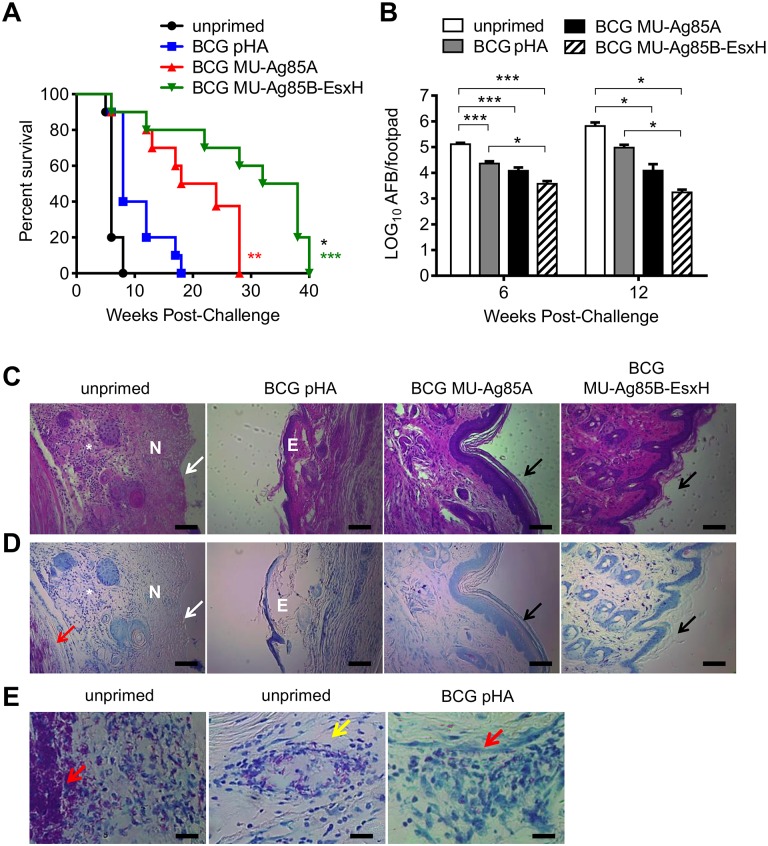
BCG MU-Ag85B-EsxH vaccination is significantly more protective than BCG or BCG MU-Ag85A against MU challenge and bacterial burden. **A**. Naïve C57BL/6 mice (black) or mice subcutaneously primed with 10^7^ empty-vector BCG (blue), BCG MU-Ag85A (red), or BCG MU-Ag85B-EsxH (green) were challenged with 10^5^ MU1615 via the footpad 8 weeks post-vaccination. At various time points, point post-challenge, the area of footpad swelling was measured. Mice were euthanized if the height of footpad swelling reached 4.5 mm. Survival curves represent time to euthanasia. Asterisk indicates statistical analysis of empty-vector BCG versus BCG MU-Ag85A (red) or BCG MU-Ag85B-EsxH (green) and BCG MU-Ag85A versus BCG MU-Ag85B-EsxH (black) by the Mantel-Cox test (n = 10 for each group). *p<0.05, **p<0.01, ***p<0.001 **B**. C57BL/6 mice were subcutaneously vaccinated as described in A (unprimed; white, empty-vector BCG; gray, BCG MU-Ag85A; black, BCG MU-Ag85B-EsxH; striped) and intradermally challenged with 10^5^ MU1615. At 6 and 12 weeks post-challenge, mice were euthanized, footpad homogenates were prepared for auramine-rhodamine staining, and acid-fast bacilli were quantified under 1000x magnification. Asterisks indicate statistical analysis by the student’s t-test (n = 16 images per group). Error bars represent standard deviation. *p<0.05, ***p<0.001 **C-E**. Tissue sections from 12 week MU-infected mouse footpads were H&E stained (row C) or Ziehl-Neelsen (ZN) stained (rows D, E) and visualized by light microscopy. Footpads from unprimed mice consistently displayed loss of epidermis (white arrows) associated with necrosis (N) and inflammatory infiltrate (*). Black arrows indicate fully intact epidermis for the BCG MU-Ag85A and BCG MU-Ag85B-EsxH groups. Edema (E) and thinning epidermis remained prominent in empty-vector BCG -primed mice but was rarely presented in either of the recombinant BCG-primed groups. Red arrows denote masses of pink ZN^+^ extracellular bacilli observed in unprimed and empty-vector-primed sections (rows D, E) and the yellow arrow highlights and granulomatous lesion in an unprimed footpad. Scale bars in rows C and D represent 100 μm (40X magnification) and in row E represent 50 μm (100x magnification).

We previously demonstrated that priming with BCG MU-Ag85A could significantly extend the survival time of MU challenged mice compared to BCG vaccination alone [15]. Upon characterizing the immunogenic properties associated with BCG MU-Ag85B-EsxH, some of which displayed enhancement over BCG MU-Ag85A, we were further interested in determining the protection profiles in similarly challenged mice. C57Bl/6 mice were subcutaneously primed with 10^7^ empty-vector BCG, BCG MU-Ag85A, or BCG MU-Ag85B-EsxH and, 8 weeks later, were intradermally challenged with 10^5^ virulent MU1615 via the footpad. The width and height of challenged footpads were measured with digital calipers during the period of infection. If footpad swelling surpassed 4.5 mm in height, mice were euthanized to reduce suffering. Fig 6A shows the time to euthanasia for unprimed and vaccinated mice. As previously demonstrated, while BCG vaccination increased the mean survival time from 6.3 weeks for unprimed mice to 8 weeks, subcutaneous vaccination with BCG MU-A85gA significantly increased survival time over BCG alone to 17.4 weeks (p<0.01). Markedly however, a single subcutaneous dose of BCG MU-Ag85B-EsxH further significantly increased the survival time of MU-challenged mice over that of BCG MU-Ag85A to a mean of 29.4 weeks (p<0.001).

Previous BU studies in mice have demonstrated a correlation between the degree to which infected footpads swell and the MU bacterial load present within challenged tissue [15, 48]. To determine if the observed enhancement of survival associated with BCG MU-Ag85B-EsxH vaccination correlated with a reduction in MU burden, mice which had received vaccinations and challenged as above were euthanized for isolation of MU1615. Infected footpads were dissected at 6 and 12 weeks post-challenge and persisting acid-fast MU in filtered footpad homogenates were stained with fluorescent auramine-rhodamine. The evaluation of bacterial load using microscopy was previously assessed to confirm similar colony forming unit (CFU) results were achieved compared to plate counting [15]. Fig 6B shows the mean acid-fast burden for unprimed mice or those primed with empty-vector BCG, BCG MU-Ag85A, or BCG MU-Ag85B-EsxH. At both 6 and 12 weeks post-challenge, all subcutaneous vaccinations resulted in a significant reduction of footpad bacterial burden compared to unprimed mice. However, priming with BCG MU-Ag85B-EsxH consistently achieved the greatest protection at both time points, conferring a significantly greater reduction in footpad bacterial replication by 1.5 log and 2.56 log at 6 and 12 weeks post-infection, respectively (p<0.001, p<0.05). Importantly, protection conferred by BCG MU-Ag85B-EsxH was also superior when compared to burdens present in empty-vector BCG (a 0.78 log and 1.7 log reduction at 6 and 12 weeks, respectively) and BCG MU-Ag85A-vaccinated groups (0.84 log reduction at 12 weeks post-challenge). This potent inhibition of bacterial growth by BCG MU-Ag85B-EsxH vaccination, as well as the intermediate inhibition by BCG MU-Ag85A correlated well with the distinct ability of each vaccine to extend survival times in the footpad challenge model.

Production of the cytotoxin, mycolactone, is known to contribute to the tissue destruction histologically observed at the foci of MU infection [49, 50]. To determine if the reduction of *in vivo* bacterial burden by vaccination correlated with protection against tissue damage, footpads were collected for histopathological analysis 12 weeks post-infection. Fig 6C displays representative images of hematoxylin and eosin (H&E) stained tissue sections from unprimed, empty-vector BCG, BCG MU-Ag85A, or BCG MU-Ag85B-EsxH primed mice. Consistent losses of epidermal layers, as well as extensive areas of internal necrosis and infiltrates of inflammatory cells were observed in footpads of unprimed mice. While BCG vaccination had a reduction in epidermal loss, substantial edema replaced necrotic lesions found in unprimed animals. However these features were rare in BCG MU-Ag85A and BCG MU-Ag85B-EsxH primed mice where, at 12 weeks post-infection, full tissue integrity remained in most animals. To visualize the organization of persistent MU bacilli *in vivo*, Ziehl-Neelsen (ZN) staining was performed on tissue sections from challenged footpads. Fig 6D displays ZN staining of the contiguous tissue sections of the above-mentioned H&E tissue sections. Large masses of pink acid-fast bacilli (AFB) present in the extracellular milieu as well as granulomatous lesions could readily be observed in tissue sections from unprimed MU-challenged mice (Fig 6D and 6E), while fewer and smaller groups of AFB were detected in empty-vector BCG groups (Fig 6E). However, AFB could not readily be observed in footpad sections from the BCG MU-Ag85 and BCG MU-Ag85B-EsxH vaccinated groups, correlating with the lower overall bacterial burden present in these tissues. Overall these data suggest that the protective BCG MU-Ag85B-EsxH immune responses, characterized by enhanced proliferation of antigen-specific Th_1_ CD4+ T cell populations and potent antigen-specific humoral IgG induction, can contribute to a reduction in bacterial burden, inhibition of tissue destruction, and an overall greater lifespan for MU1615-infected mice, compared to BCG and BCG MU-Ag85A.

## Discussion

Buruli ulcer is a neglected tropical disease whose persistence continues to inflict severe patient morbidity in the absence of rapid diagnosis and treatment. Today’s diagnostics are limited to molecular techniques, microbial culture, or histopathological analyses not readily available in the most afflicted regions. Currently, the standard of care involves lengthy medical regimens, including rifampin and streptomycin treatment, both of which may confer issues of toxicity. Generation of a prophylactic vaccine against *M*. *ulcerans* infection could be incredibly invaluable, especially due to the disproportionately high incidence of BU in children, the threat of long-term disfigurement, and associated social stigma. Despite many efforts to develop an efficacious BU vaccine, new candidates have either not displayed immunity or have conferred limited and short-lived protection against experimental MU infection.

However, we recently have shown that a single subcutaneous dose of quality controlled BCG-based vaccine overexpressing the MU mycolyl transferase antigen 85A could significantly decrease bacterial burden, pathology, and increase survival time following MU1615 footpad challenge [15]. These protective effects were significantly greater than those conferred by the previously most protective vaccine strain to date, *M*. *bovis* BCG. Interestingly, a subsequent study highlighted MU-Ag85A as conferring protection when expressed in the *M*. *marinum* genetic background as well [51]. With the successful progress of recombinant BCG and *M*. *marinum* expressing MU-Ag85A as BU vaccine candidates, we decided to further improve a candidate vaccine strain by expressing other known immunodominant antigens. To this end, a novel recombinant BCG strain expressing the MU-Ag85B-EsxH fusion protein was generated, frozen into quality controlled accession lots, and evaluated for immunogenicity and protection against experimental BU in the mouse model.

Several aspects of immunogenicity were investigated in the present study, including the induction of MU antigen-specific CD4^+^ T cell and memory populations, the production of IFNγ-secreting cells responsive to stimulation by whole MU and cellular components, and the production of the antigen-specific humoral responses. Interestingly, both BCG MU-Ag85A and BCG MU-Ag85B-EsxH were equally more significantly immunogenic compared to BCG when examining adaptive T cell responses and memory populations. In contrast, BCG MU-Ag85B-EsxH vaccination was capable of inducing significantly greater IgG responses to both rAg85A and rAg85B compared to empty-vector BCG at multiple time points post-vaccination, while inoculation with BCG MU-Ag85A was not. Although the importance of humoral responses for immunity against MU is not well characterized, antibody mediated protection might be of major relevance against advanced stages of MU infection, where bacilli are predominantly found as extracellular clusters. Furthermore, previous studies have demonstrated the potential efficacy of antibody-based therapies against experimental *M*. *tuberculosis* infection [45–47].

In addition to an evaluation of immunogenicity for the BCG MU-Ag85B-EsxH vaccine, we were interested in characterizing any improvement in protection during *in vivo* MU challenge compared to empty-vector BCG or BCG MU-Ag85A. Vaccination with a single subcutaneous dose of BCG MU-Ag85B-EsxH significantly increased the lifespan of infected mice over that of unprimed mice or empty-vector BCG by 4.7-fold and 3.7-fold, respectively. Strikingly, expression of Ag85B-EsxH in BCG also conferred significantly greater protection compared to BCG MU-Ag85A by increasing the mean survival time 1.7-fold compared to this strain. This result was also associated with a significant decrease in MU burden at 6 and 12 weeks post-challenge, with BCG MU-Ag85B-EsxH reducing bacterial burden compared to empty-vector BCG and BCG MU-Ag85A by 55-fold and 7-fold at 12 weeks, respectively.

Of note, we were unable to demonstrate antigen-specific immune responses to the MU-EsxH protein. Proper molecular mass and plasmid sequencing of the fusion protein were confirmed in the BCG MU-Ag85B-EsxH accession lot according to our previously published quality control protocol, suggesting that the assays chosen were either not sensitive enough to detect EsxH immune responses or could not detect responses to EsxH as a fusion to Ag85B. Alternatively, the immunogenicity reagents used may have not represented a high enough homology to the MU-EsxH protein sequence. The TB10.4 MHCII tetramer, TB10.4 ELISPOT peptides, as well as recombinant TB10.4 protein used in the IgG ELISA were of *M*. *tuberculosis* origin, which shares 84% amino acid sequence identity with MU-EsxH. These reagents were chosen because of their ready availability from BEI resources; however, further characterization of antigen-specific immunity to the TB10.4 homolog will require the use of EsxH-specific reagents. This does insert a degree of complexity when determining the degree of contribution to protection each antigen provided, or if either antigen alone could singularly be responsible for the increase in protection observed in the present study. This would require a comparative analysis with BCG MU-Ag85B or BCG-EsxH, which is planned for future investigations.

Previous vaccine studies which utilized Ag85 members and TB10.4 in tuberculosis models may shed light on their relative contributions towards immunity against MU. A recombinant strain of BCG which overexpressed the *M*. *tuberculosis* Ag85B was previously shown by Horwitz *et al*. to significantly reduce bacterial burdens in both the lungs and spleens of challenged guinea pigs compared to either recombinant Ag85B protein or BCG vaccination alone ([31, 37]). Mu *et al*. generated recombinant adenovirus vaccines which expressed *M*. *tuberculosis* Ag85A or the fusion between Ag85A and TB10.4. Following vaccination and subsequent challenge with H37Rv, bacterial burden in the lungs of the antigen fusion strain was significantly reduced compared to the monovalent adenovirus-Ag85A and about 10 fold reduced compared to BCG alone [52]. Similarly, purified recombinant *M*. *tuberculosis* Ag85B or TB10.4 used as monovalent protein vaccinations was shown by Dietrich *et al*. to decrease bacterial burden in the mouse model of tuberculosis; furthermore, vaccination using either a mixture of the two or a fusion of the antigens significantly further reduced bacterial loads [34]. Interestingly, the fusion strategy for two MU proteins may itself confer an immunological advantage over separately expressed antigens, as shown by Palendira *et al*. [53]. In this study, vaccination with BCG separately overexpressing both *M*. *tuberculosis* Ag85B and the immunodominant *M*. *tuberculosis* antigen, ESAT-6, was not as efficacious as a BCG strain encoding a fusion between the two antigens in reducing bacterial burden.

Future improvements in strategies for BU vaccine design could be made to increase the protective qualities demonstrated by rBCG. We have shown that expression of MU-Ag85A or MU-Ag85B-EsxH both confer protection against the effects of MU infection, suggesting that future studies may have success in designing BCG strains which express a combination of these antigens or novel immunodominant MU proteins. Indeed, Sun *et al*. previously demonstrated that a triple antigen encoding strain of BCG (AERAS-402) which expressed *M*. *tuberculosis* Ag85A, Ag85B, and TB10.4 generated stronger immune responses and conferred significantly improved survival compared to BCG vaccinated mice when challenged with a hypervirulent *M*. *tuberculosis* strain [36]. In addition to improvements in priming strategies, boosting with recombinant protein or other recombinant mycobacterial or viral vectors may also enhance vaccine efficacy. We previously demonstrated that boosting rBCG with recombinant *M*. *smegmatis* significantly improved protection in the mouse model of BU [15]. Furthermore, human clinical trials have effectively used recombinant modified vaccinia virus Ankara (MVA) expressing Ag85A to boost BCG by inducing potent and durable Th1-type responses as well as high levels of antigen specific T cell populations [30]. This approach has also recently been successful in boosting the triple antigen AERAS-402 strain of BCG to generate antigen-specific CD8^+^ T cells which produced high levels of anti-mycobacterial IFN-γ, TNF-α and IL-2 [35].

Use of mycolactone negative mutants or other attenuated MU strains may represent an alternative approach as well. There is experimental evidence to support this strategy, whereby subcutaneous vaccination with the mycolactone negative strain, MU5114, yielded a delay to footpad swelling similar to that induced by BCG vaccination [41]. Furthermore, species of mycobacteria possessing greater genetic homology to MU than BCG may represent a richer source of protective antigens and lack the potential pathological features of an MU-based vaccine. Indeed, we and others have previously demonstrated that subcutaneous vaccination with *M*. *marinum* was significantly better able to delay the pathology of MU infection versus BCG alone [51, 54]. Further novel attenuated and immunogenic MU strains, or strains with high genetic similarity to MU, may hold potential as priming or boosting agents to recombinant BCG.

The well-supported safety profile, an established global administration infrastructure, reasonable cost of production, and previously demonstrated protection support BCG as an ideal vehicle for BU vaccine design. Further discovery of MU-specific antigens could be an invaluable source of protective immunogens and, upon combined expression by BCG, may achieve complete protection against experimental MU infection. This, in addition to identification of cross-reactive antigens which confer the intrinsic protection observed with BCG, will be essential to the application of this ubiquitous vehicle as an efficacious and safe Buruli ulcer vaccine.
